# The Role of Information Systems to Manage Cerebral Palsy

**Published:** 2016

**Authors:** Sima AJAMI, Ali Akbar MAGHSOUDLORAD

**Affiliations:** 1 Department of Health Information Technology and Management, School of Medical Management and Information Sciences, Isfahan University of Medical Sciences, Isfahan, Iran.

**Keywords:** Cerebral Palsy, Information System, Registry, Rehabilitation, Minimum Data Set

## Abstract

**Objective**

In healthcare system, it is necessary to have exact and accurate information in order to address health care needs and requirements of society members as well as expectations of policy makers, planners and decision makers. The aim of this narrative review article was to explain the role of information systems in cerebral palsy management and identify the advantages and barriers to the development of cerebral palsy registry system. Data were collected using databases such as of Science Direct, PubMed, Proquest, Springer, and SID (Scientific Information Database). Overall, 65 sources were selected. One of the biggest challenges for children with physical and motor disabilities in rehabilitation center is access to a system, which provides a comprehensive data set reflecting all information on a patient’s care. Thus, data and information management in children with physical and motor disability such as cerebral palsy facilitates access to data and cerebral palsy data comparison as well as the monitoring incidence rate of cerebral palsy, enhancing health care quality; however, there are always numerous barriers to establish the system. One of the ways to overcome these problems is the establishment of a standard framework of minimum data sets and exact definition of its data components. Reliable standards in the use of applications as well as user-friendly software will ensure patients’ data extraction and registration.

## Introduction

Cerebral palsy (CP) is the most common chronic motor disability and neurological complication in children, which happens because of a non-progressive lesion in the developing brain (1). This lesion may occur before, during, or after birth and is usually accompanied by sensory, perceptive, cognitive, communicative, and behavioral disorders as well as epilepsy, muscular-skeletal problems all of which lead to limited activities and reduced social participation (2-4). The rate of CP incidence has been 2-3/1000 births in Europe in 2000 (5-7). Despite technology improvement in the field of neonatal intensive care and prenatal care in current decades, it is still one of major and common causes of childhood developmental disorders (7). CP is not a new disorder; but a brain disorder, normally appears as an abnormality in movements, tonicity, and posture, which may be present with other disabilities and defects. It can sometimes be considered as a group disease and affect the power of movement, learning, hearing, vision, and thinking. Most brain growth is up to two yr and brain injury during pregnancy (fetal) until 2 yr after birth called CP. However, eventual age for CP diagnosis is 3 or even 5 yr old and the development may be placed in different categories from a diagnostic point of view (8).

Physical development of children with CP is one of the most complex problems that limit a wide range of motor activities in these children. Dysfunction in various motor system function in children with CP, leads to reduced work capacity of upper limb, limited weight tolerance and transmission functions of lower limp, limited dynamic and static functions of backbone all of which eventually lead to limited bioenvironmental abilities and social adaptation challenges. Poor motor ability in children with CP ultimately has negative impacts on all developmental aspects. Although the rate of brain injury is fixed and the disease does not progress, the disorder undergoes some changes (9-12).

The CP information system (CPIS), such as other information system (IS), depends on identification, collection and processing of data for producing useful information. Lack of the integrated IS for collecting standard data causes undesirable effects on exchanging, comparing, and managing. Nowadays information management system condition in most of developing countries is not encouraging. To implement IS, all related and required patient information must be available (12). “Lack of methods and technologies for collecting internal and standard data causes great gaps and suppress the ability in interchange data and also internal interoperability with other ISs”(13).

“Inappropriate information scattering makes undesirable effects on patients’ future and preset care and so burdens more expenses on system”(13). “Lack of integration among ISs is a barrier versus systematized analysis guidance on health system”(13). Data collected without structured contents do not promote knowledge level.

“When data elements are gathered from different sources, they should be put under some regulations and standards for integrated maintenance”(13). “Nature of chronic disease, which needs information from several providers at the same time, and also a patient’s need for accessing in his clinical information have made the creation of the integrated IS necessary. The IS is an unavoidable necessity for huge investing and planning in order to quantitative and qualitative promotion of services offer, studying of services effectiveness amount in treatment performance, and making perseverance in case process. Experts believe that ISs in both health services management field and implementing care processes, make it possible to compare different course performance”(13). This system plays an important role in effectiveness evaluation and appropriate decision-making (12-19).

CP information will properly affect rehabilitation management if policy makers in every part of management system use the provided data. Decision making includes determining the status, identifying priorities, and implementing planned activities.

Rehabilitation professionals collect and exchange CP information among organizations and people using standard tools and in a single language. Such tools facilitate communication and ensure effective actions of people, clinics, and rehabilitation organizations involved in the health care process of CP patients. Moreover, demands of service providers of insurance organizations will be addressed easier and faster (8, 12, 20-22). 

The aim of this study was to determine the role of IS in CP management, outcomes, and barriers to establish CP registry system.

## Materials & Methods

The present narrative review article was performed to identify the role of IS in CP management. It was divided into three phases as literature collection, assessing, and selection. Data were collected through databases such as of Science Direct, PubMed, Proquest, Springer, and SID (Scientific Information Database). 

The search was performed in early May in 2013 until September 2014. We employed the following keywords and their combinations: cerebral palsy, health care information system, registry, and minimum data set in the searching areas of titles, keywords, abstracts, and texts from 1982-2014. Overall, 170 articles were collected, of which 65 cases were selected based on their relevancy.

## Results

In the new millennium, people face new challenges in health care, such as the growing trend of non-catching diseases. With increasing pressure to reduce costs, improve quality and effectiveness, the need for health care data is felt more than ever (23).

Managers and health care providers should have enough knowledge on the management of health IS to enhance the efficiency and effectiveness of the organization because access to reliable, timely, accurate, and exact information is the basis of decision making, policy making, and planning at different management levels (24). Information management uses standard tools and a single language in data collection and exchange among organizations and people. These tools facilitate communication among people and organizations involved in health care and improvement of service quality for patients (22, 25-26). Development of information and data sets is essential for the establishment of health care IS, which concentrate on concepts associated with diagnosis, intervention, and outcomes of diseases (27-28). In health system, it is necessary to have access to exact and accurate information for policymaking, planning, decision making, and addressing health requirements and needs of the society. It is also essential to develop IS and use the best available data in order to provide on time and practical information for decision making. Moreover, IS leads to less cost, higher quality of health care services, and better organizational reputation (23).

Health information is a set of processed data valuable for supporting clinical decisions. The information is used by providers of health care services for disease diagnosis, improvement of health care programs, evaluation of service effectiveness, and identification of outcomes and prognosis; therefore, designing and implementing an efficient information system is one of health system priorities (29-31).

The most important part of information management system is data collection considered as the basic tool for a detailed description of the health care process, evaluation, diagnosis, interventions, outcomes, and documentation. Managers need reliable and accurate data for resource allocation and management. The first step toward data standardization in order to facilitate, share, and compare it among different centers is the establishment of a minimum data set (27, 32).

Establishment and development of national MDS in long-term care services will lead to standardizing of data, and data content, as well as, a common language in data exchange, which eventually leads to better quality and efficiency of health care services (22). MDS is a standard tool for data collection, which guarantees access to exact and clear health data. It is also considered as a conceptual framework and the basis for access to effectiveness indicators (31-34).

In developing countries all data related to patients is placed into a national MDS to be accessible for auditing, analysis, and investigation of data quality and in countries such as America and Canada Ministry of Health uses information network to access national MDS (30, 35). 

Standard data on CP patients is an essential tool for the representation of information on services provided and an image of patients’ conditions, used to describe clinical features, the severity of defects and damages, the status of health care services, and implementation of health care guidelines compared to existing standards (21). The aim of CPIS is a collection of the most exact and comprehensive data for different applications such as: CP monitoring, identifying interventions, which affect quality of life, identification of treatment methods, and evaluation of preventive strategies for patients and their families’ future. Use of IS in the care and treatment process of CP children enables families to share information among members and health care team (8, 36). Public and private health care system of the United States has established especial rules and regulations for the promotion of services to CP patients. For example, a 15-yr old child’s MDS with CP diagnosis should be registered in seven different databases (electronic medical records, clinical databases for neonatal intensive care, early intervention records, childhood and school records, outpatient therapy sessions, early medical records, specialist records, and programs for children with specific health care needs). Most countries with a high percentage of CP have global health care systems for development and maintaining CP data. Data collected from different CP patients can play an important role in health care planning and even the age of CP children is important in data collection (8, 21, 37).

**Fig 1(SA):**
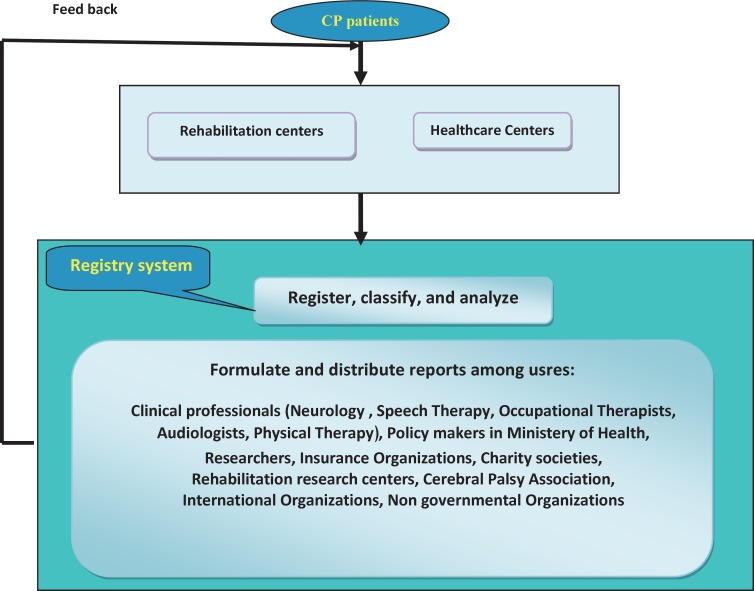
The process of establishing CP registry system

To improve patients, health care activities should concentrate on real needs and problems. Decision making on health strategies and interventions should be based on timely and reliable information on disease distribution, which can be only obtained through a proper IS. A database for collection, processing, and distribution of data in the form of information or management of data associated with the process or incidence of diseases guarantees design and implementation of information management systems (19, 38). Improvement in care system quality, identification of groups exposed to disease risk, codification of control programs, prevention and evaluation of diseases is possible if a national IS is created for every disease and the related data is collected completely and timely (39). This data can be used in planning, implementing, evaluating public and clinical health activities, so the only way to achieve a better treatment is continuous, and regular data collection. As a result, it is essential to have an information bank for collection, process, and distribution of data in the form of information or manages data related to the process or incidence of diseases. It is necessary to design and implement a population-based IS, which leads to a significant revolution in health research domains (23, 40, 41). Moreover, in healthcare industry a large amount of data is collected from physicians’ offices, hospitals, rehabilitation centers, and outpatient clinics whose goal are to facilitate the care process, management of patients, reimbursement, clinical research, and development of health care policies (Figure 1) (42).

**The advantages of implementing CP information system**

Lack of a comprehensive model of rehabilitation leads to lack of MDS and comprehensive standards. In fact, one the greatest challenges regarding physical and motor disabilities of children in rehabilitation centers is access to a system which provides a comprehensive data set reflecting all health care activities related to the patient (8, 20, 43). Therefore, data and information management in children with physical and motor disabilities such as CP facilitates access to data, CP data comparison, and monitoring the incidence and prevalence of CP (43-45). Other advantages include: assessment of CP causes based on neurological subgroups and birth conditions (20), timely and accurate decision making, reduction and control of costs (23), improvement of cost effectiveness, enhancing health care quality, correction and facilitating of processes, avoiding duplicate tests and procedures, gaining a higher understanding of CP causes, quality assurance, evaluation of preventive strategies, management as well as time and cost saving, respect to data confidentiality, timely and continuous service providing, and assistance in planning services to CP patients (23, 46-48). Moreover previous studies (33, 49, 50) introduce the standard minimum data set as a tool, which facilitates access to evaluation results, coordination and establishment of consultation culture, and prevention of medical errors and deductions. In addition, an educational instrument helps legal research and clinical assessment (49, 50). Health care members can share information through a framework, created for research. Tracking and follow up procedures, including tests, diagnoses, and medical care in the treatment and evaluation of CP may be expensive and time consuming, but it is feasible through management and maintenance of CP data. Intervention evaluation in CP children is made based on the severity of motor impairment (8).

**Barriers to the creation and implementation of CP information system**

Today, due to the increasing need for health IS, their increasing complexity, and significant diversity and innovation in supplying these systems, they have become one of concern in the health sector. Barriers, which lead to lack of development of health information system, are divided into personal and environmental factors (51-53). Environmental barriers include: lack of comprehensive utilization of developed management systems and an integrated, logical relation among service providing sectors, lack of binding regulations and rules related to health management system, low quality of information, lack of integration of the health system (51, 53-56), lack of participation of private sector in research, resistance to change, lack of strategies for organizational information development (54), lack of access to hardware infrastructures and limited access to applications, high costs of access to technology and required tools, lack of access to sufficient information for implementation of information systems (23, 52, 57), lack of informatics standards and data dictionaries for implementation of health systems, lack of protection of information confidentiality, lack of continuous monitoring, lack of training courses, lack of investment and financial support for developments of health IS (52, 53, 58-60). 

Personal barriers include: lack of managers’ adequate knowledge on importance of statistics and information, lack of professionals’ incentive to participate in patient-based interactions (23, 59), carelessness and negligence in data collection, error in registration, lack of patients’ awareness of treatment methods conducted virtually, staff resistance to changes, lack of management belief in staff abilities to work with information systems (51, 52, 57, 61), lack of sufficient financial incentives for private sector, low general literacy of patients regarding their disease and health, lack of skills, facilities, and patience for adequate data collection and registration (23, 62-63).

**Proposed solutions to address barriers to the creation and implementation of CP integrated information systems**

Supervision and evaluation are among important management tools in every IS, which lead to higher quality of services and improve managers’ attitudes toward the use of information. These tools help in avoiding duplicate registering and creating a unique Identification (ID) for every patient as well as exact and timely registries of patients’ data (33, 47, 56). Some solutions include development of a standard framework for minimum data sets and exact description of its data elements, ensuring data security and confidentiality (57, 64-65), increasing interaction between public and private health care providers. Promoting correct and optimal use of computer and communication technology, particularly internet and statistical software, holding more training courses in order to increase staff computer knowledge, use of reliable standards for different software applications, use or user-friendly software able to register and extract data (23, 52, 54, 56), facilitating the use of IS that all users can enter and restore data based on standards and legislations regarding data confidentiality (56, 57, 59).

**In conclusion, **CP information system has many advantages such as: easier access to health information, access to patients’ integrated information, spending less time by both patients and staff, less duplicate registry and tests, lower costs, improvement in health care quality, facilitating research in health domain, providing a clear and uniform definition of data elements, monitoring disease progress and identifying influencing factors, easier data retrieval and extraction of diseases statistics. It also underlies an appropriate CP information system (personal health record or electronic health record) which can monitor the treatment of CP patients continuously. 
